# Evaluating the efficiency of public policy measures against COVID-19

**DOI:** 10.3906/sag-2106-301

**Published:** 2021-12-17

**Authors:** Rahmet GÜNER, İmran HASANOĞLU, Firdevs AKTAŞ

**Affiliations:** 1 Department of Infectious Diseases and Clinical Microbiology, Yıldırım Beyazit University School of Medicine, City Hospital, Ankara Turkey; 2 Department of Infectious Diseases and Clinical Microbiology, LÖSANTE Children’s and Adult Hospital,Ankara Turkey

**Keywords:** COVID-19, prevention, public policy, contact tracing, lockdown, vaccination

## Abstract

The World Health Organization (WHO) declared COVID-19 as a Public Health Emergency of international concern on January 30, 2020, and a pandemic on March 11, 2020. Afterward, it spread rapidly all over the world, causing almost 4 million deaths as of June 2021. It is clear that effective preventive measures are needed in this devastating disease, which still has no cure. In addition to mask using, social distancing, and hygiene practices, which enter our lives as the most basic precautions, communities aim to reduce the effects of the COVID-19 pandemic. All over the world, the measures taken and activities performed in the COVID-19 pandemic are discussed, and information in this regard is shared. Mask usage, social distancing, hygiene, avoiding crowded and closed areas, contact tracing, rapid and accurate testing, increased indoor air quality, vaccination, and lockdown measures constitute the main preventive measures. This review summarizes the efficiency of public policy measures against COVID-19.

## 1. Introduction

**Figure 1:**
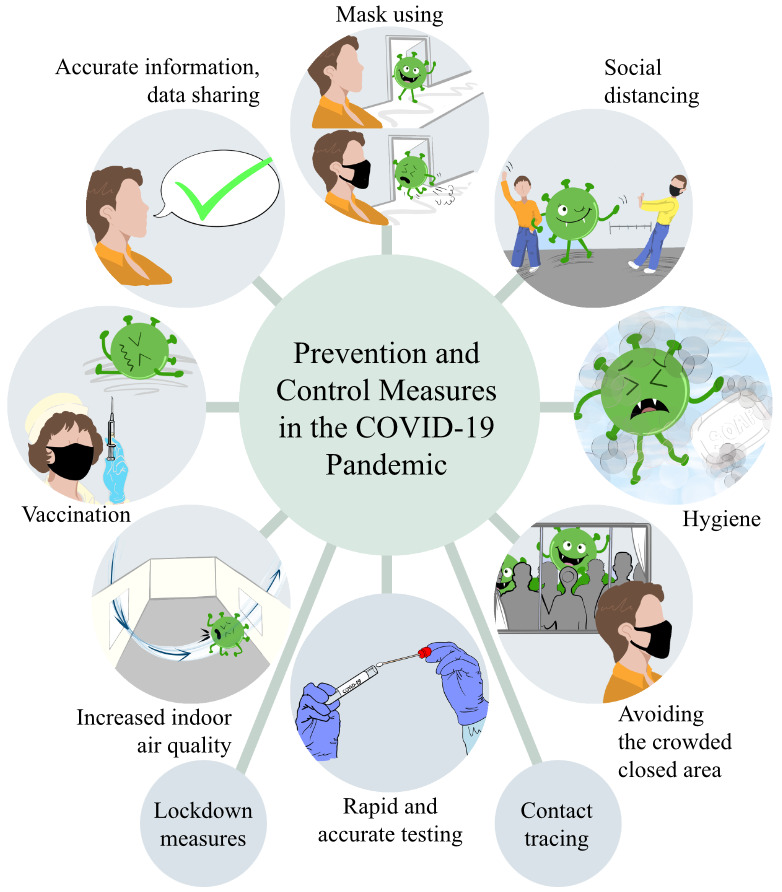
Prevention and control measures in the COVID-19 pandemic, courtesy of Özlem Özkan (architect, medical illustrator).

The preventive measures to be taken in the fight against the COVID-19 pandemic and their effectiveness have attracted attention as much as the agent and its treatment. The transmission routes of SARS-CoV-2 are particularly close contact, respiratory droplets, and in some cases, airborne transmission. To protect from the deadly and devastating effects of the pandemic, measures have been taken at the social level in many countries. The most basic of these measures is the use of masks, social distancing in crowded environments, and keeping hygiene; especially hand hygiene is a priority. Considering the changing social characteristics of each country, it has become inevitable to go into isolation especially in societies that are culturally socializing or in places where population density is high. Air transportation, which almost removed the borders between countries in the world of the 2000s, played an important role in the spread of the SARS-CoV-2 between continents within hours. Island countries and countries with good border control had an advantage in preventing the spread of the disease through their borders. In this review, data on the effects of preventive measures taken at the community in the management of the COVID-19 pandemic are discussed. Measures with demonstrated effectiveness are given in Table 1 and Figure 1.

**Table 1:** Prevention and control measures in the COVID-19 pandemic.

Mask using
Social distancing
Hygiene
Avoiding the crowded, closed area
Contact tracing
Rapid and accurate testing
Increased indoor air quality
Vaccination
Accurate information, data sharing, and effective field studies
Lockdown measures

### 1.1. Mask use, social distancing, eye protection, and hygiene efficacy of prevention of COVID-19

Conclusive evidence obtained by cluster reports suggests the principal transmission path to be respiratory, with proximity and ventilation as the most important factors determining the risk of transmission. Although there are few cases where direct contact or fomite transmission are suspected, none have decisively eliminated respiratory transmission.

The main modes of transmission of SARS-CoV-2 are droplet, contact, and airborne. Since the SARS-CoV-2 virus particle is very small (0.08–0.14 μm), transmission can also occur under certain conditions where aerosols are produced. Since droplets can reach the mucosal surfaces of a susceptible person from a distance of less than 1 m, the transmission may occur through the respiratory tract through face-to-face talking, breathing, coughing, and sneezing during daily activities. Droplets can spread to a volume of radius exceeding 1 m by airflow, and these aerosols can survive for a long time in the environment [1]. 

In a contact tracing study of the COVID-19 outbreak from public transport in China’s Huanan Province, 10 people were found to be infected from a single COVID-19 case. The first index case traveled without a mask for 2.5 h by bus and then for 1 h on a smaller bus. A total of 10 people were found to be infected on the first bus although 7 of them were not in direct contact with the index case [2]. This epidemic encountered in public transportation is very important in terms of showing the increasing probability of transmission in closed and crowded environments.

The role of asymptomatic cases in the transmission is one of the biggest obstacles in limiting the spread of the disease. Clusters of COVID-19 cases have been reported in relatively limited and public places such as shopping malls, workplaces, buses, ships, prisons, hospitals, and nursing homes. A total of 40 cases of COVID-19, accounting for 75% of the 53 definitively diagnosed cases reported from the Baodi region of Taijin, China, were associated with store exposure. Of these 40 cases, 6 (15%) were store employees, 19 (47.5%) were customers, and 15 (37.5%) were close contacts (secondary cases). Three cases that developed after the first case were working despite the symptoms [3]. COVID-19 cases resulting from community gatherings have been reported from many countries. The presence of large numbers of passengers, narrow spaces, and common surfaces can turn public transport into an epidemic-spread center [2–4]. For this reason, in most of the countries, there has been a tendency towards measures such as limited use of public transport vehicles and capacity reduction in passenger buses.

While it is a consistent recommendation for symptomatic individuals and healthcare workers to use masks, inconsistencies were observed at the beginning of the pandemic regarding the use of masks in the community [5]. However, in the later stages of the pandemic, WHO renewed its recommendations and recommended the use of masks in the community World Health Organisation (2019). Mask use in the context of COVID-19 [online]. Website https://apps.who.int/iris/handle/10665/337199 [accessed 15 June 2021].. In fact, many public health experts and epidemiologists have argued that the use of masks in the community should be made compulsory, since it slows down transmission.

The effectiveness of mask use is based on some important facts given below:

• SARS-CoV-2 is a virus transmitted from person to person,

• Respiratory droplets act as the main transmission route,

• Mask usage reduces the possibility of transmission by reducing the viral load,

• Covering the mouth and nose with a mask reduces the amount of virus-carrying droplets.

Although several studies have attempted to indirectly assess the effectiveness of masks against the spread of various respiratory infections, few published randomized controlled trials have advocated the use of masks as an effective strategy for controlling SARS-CoV-2 transmission. In the randomized double-armed DANMASK-19 study conducted in Denmark, in which 6000 people participated, people who spent more than 3 h a day outside the home were grouped as 1:1 mask users / nonusers. With a 30-day follow-up, the development of infection and the formation of antibodies in the serum were planned by PCR in both groups. Infection development (1.8% vs 2.1%, respectively) in the group using and not using masks (95% CI, –1.2 to 0.4 percentage point; p = 0.38) did not differ statistically. However, the study has limitations such as inconclusive results, missing data, variable compliance rates, and patient-reported findings with home tests. Therefore, it would be correct to interpret the study as showing a low level of protection, which did not reduce the SARS-CoV-2 infection rate by more than 50%, that was conducted in a country with a moderate infection rate with a community adhering to social distancing and generally wearing masks albeit not fully widespread [6]. 

In a metaanalysis, Chu et al. evaluated the effectiveness of social distance, face mask, and eye protection both in health institutions and in society. A total of 172 studies from 16 countries from 6 continents were included in the metaanalysis, some of which were conducted in health services and some in the community. Social distancing of 1m or more, mask use, and eye protection were associated with lower infection rates [7].

A systematic analysis of studies reported from low and middle-income countries was conducted to evaluate the effectiveness of community-based interventions. One of the 17 studies meeting the evaluation criteria was from Africa (Madagascar), and the rest were from Asia 9 (China 5, Bangladesh 2, Thailand 2), South America 5 (Mexico 3, Peru 2), and Europe 2 (Serbia and Romania). According to the results of the metaanalysis, it was possible to prevent Severe Acute Respiratory Syndrome (SARS) and influenza infections, which use similar transmission routes, with the use of face masks and hand hygiene practices. The researchers emphasized that it would be possible to control the epidemic by increasing the level of compliance with the basic measures that can be taken in low and middle-income countries [8].

In a systematic review and metaanalysis on the effectiveness of social distancing, mask, and eye protection, it was determined that a factor affecting the compliance of people with the rules of physical distance was the incidence of SARS-CoV-2. A higher rate of compliance was achieved with the increase in the number of people who got infected. It has been shown that social distance of more than one meter is likely to result in greater reductions in viral infection, with each 1-m increase in distance having an approximately 2 times higher relative effect. The effectiveness of masks and eye protection has been emphasized based on studies on MERS-CoV and SARS-CoV [9].

In a mathematical modeling study conducted in India, it has been shown that the spread of the COVID-19 can be brought under control if policies regarding rapid testing, contact tracing, and mandatory use of masks and gloves are implemented in the 3 states most severely affected by the pandemic [10]. South Korea is among the countries that have best controlled the COVID-19 pandemic. When the reasons are examined, it seems that the widespread acceptance of the use of masks in the community from the very beginning seems to be one of the most important contributing factors. In addition, an aggressive “trace, test, and treat” program was implemented in South Korea. The public was asked to avoid large gatherings and crowded places, and to follow quarantine protocols such as wearing masks, hand washing, and social distancing [11]. South Korea compared the measures taken to reduce the number of cases in the COVID-19 pandemic and 8 infectious respiratory diseases with previous years. With their analysis, they showed that not only COVID-19 but also other infectious diseases using similar modes of transmission decreased significantly regardless of age and gender [12].

In Ontario, Canada’s most densely populated province, a weekly decrease of 22% was observed with the introduction of mask use. In addition to the mandatory mask usage, closely monitoring the mobility of people and the increase in cases with an application such as Google geo-location data will enable early identification of the problem. With the survey conducted in Canada, they also determined that mandatory mask usage brought a 27% increase in compliance in public places. It was concluded that the mandatory wearing of masks in closed public places is a powerful tool to limit the transmission of COVID-19, especially given its relatively low cost to the economy [13].

### 1.2. Use of accurate and rapid diagnostic tests

Rapid diagnosis and early isolation of the infected patients have a crucial role in the prevention of SARS-CoV-2. It is very important that the genetic material of the SARS-CoV-2 virus was obtained in China in about three weeks. In the case of SARS-CoV-1, this period was approximately 3 months. South Korea is one of the countries that widely use diagnostic tests that give fast and accurate results. With the increasing number of suspected and/or symptomatic patients to be tested for COVID-19 in Korea, a safe and efficient screening system has been established. The recommended nasopharyngeal swab procedures for the detection of SARS-CoV-2 are aerosol-generating. It requires special rooms such as isolation rooms that ideally require disinfection and ventilation between patients. Drive-through screening (DTS) centers have been implemented by hospitals and local authorities to facilitate sample collection due to the lack of testing capacity in these areas [14]. As the whole procedure takes about 10 min per person and several people can be served in different steps at the same time, the testing capacity has increased significantly. DTS centers contributed to the early diagnosis of patients without delay. In addition to the commonly used measures, Korea was able to effectively reduce the number of cases that had a sudden increase with this application [15].

### 1.3. Contact tracing and isolation measures for prevention of COVID-19

Contact tracing is the process of identifying, evaluating, and managing people who have had contact with an infected symptomatic or asymptomatic person. This method, which is also adapted to COVID-19, ensures that the identified contacts are quarantined and prevents the increase of transmission between people. It is an important public health tool. Contact tracing can also help people who are at higher risk of developing serious illness to be diagnosed at an early stage. It may be possible to break the chain of transmission with rapid diagnosis, isolation, and quarantine. While new variants of the virus are more contagious, thorough and timely contact tracing and quarantine of contacts allow controlling the spread of the virus World Health Organization (2021). Coronavirus disease (COVID-19): Contact tracing [online]. Website https://www.who.int/news-room/q-a-detail/coronavirus-disease-covid-19-contact-tracing [accessed 15 June 2021]. [16]. Mathematical models made for this purpose have an important role in being prepared for infectious diseases. In particular, it allows health policymakers to plan for potential issues regarding public health before they occur. Similarly, in Turkey, keeping the number of cases under control was aimed by utilizing early isolation with contact tracing methods and quarantine measures with a specialized unique code, called LFH (Life Fits Home), assigned to every individual that allows central tracking of contacts through QR codes and mobile applications Republic of Turkey Ministry of Health (2021) COVID-19 Information Page [online]. Website https://covid19.saglik.gov.tr/TR-66339/temasli-takibi-salgin-yonetimi-evde-hasta-izlemi-ve-filyasyon.html [accessed 15 June 2021]., European Centre for Disease Prevention and Control (ECDC) (2020). Contact tracing: public health management of persons, including healthcare workers, having had contact with COVID-19 cases in the European Union [online]. Website https://www.ecdc.europa.eu/en/covid-19-contact-tracing-public-health-management [accessed 15 June 2021].. Based on the SARS experience, countries such as Singapore and Taiwan identified close contacts in COVID-19 infection and quarantined them for 14 days from symptom-free contact. It aimed to diagnose contacts with symptoms early with PCR tests [17,18]. A modeling study by Hellewell et al. showed that a new COVID-19 peak can be controlled within 3 months with effective contact tracing [19]. The strict contact tracing strategy of Turkey is a good example of this. For instance, with this method, early detection of 23 PCR (+) cases, 14 of which required hospitalization, was made possible [20]. For this purpose, the use of artificial intelligence and computer technologies will provide data that will contribute significantly [21,22].

### 1.4. Increased indoor air quality

At the beginning of the COVID-10 epidemic, the most notable transmission routes were close contact with an infected person and contact with virus-contaminated surfaces. However, airborne transmission has been much debated. Especially procedures that generate aerosolization increase the risk of transmission. A systematic review and metanalysis of airborne transmission of SARS-CoV-2 in indoor environments evaluated 14 studies. According to the findings, the possibility of airborne transmission of SARS-CoV-2 in closed environments is very high. It is possible to improve indoor air quality and, therefore, reduce the possibility of transmission, by improving ventilation and keeping an interpersonal distance of more than 2 m, especially in hospitals and crowded places [23]. SARS-CoV-2 is spread through respiratory droplets in a confined space, especially in a building, by close person-to-person contact. In a study evaluating environmental factors affecting the transmission of SARS-CoV-2, it was shown that SARS-CoV-2 can remain on the surfaces of fomites for at least 3 days, depending on the conditions. When SARS-CoV-2 is aerosolized, it is stable for at least a few hours [24,25]. SARS-CoV-2 is rapidly inactivated on surfaces exposed to sunlight. It has been shown by case clusters that the probability of transmission increases in a closed, crowded, and poorly ventilated indoor environment. While uncertainty regarding aerosol transmission remains, focusing on poorly ventilated confined spaces, crowded areas with large numbers of people, and close contact situations is crucial to prevent the spread of COVID-19 [26,27]. It was also stated that strategies to improve indoor air quality should form the basis of future building designs. It is very important to improve indoor air quality with methods that include engineering, design, and air disinfection technologies through interdisciplinary collaborations [28].

### 1.5. Vaccines and their efficacy in stopping the COVID-19 pandemic

In infectious diseases, if the number of individuals who have become immune to the disease in the community is above a certain rate, the spread of that disease will decrease significantly. Vaccination is the most effective method to protect people and stop the pandemic, as well as prevent variant development. Because, as the virus spreads among humans, it changes as it continues to multiply, and more contagious, more severe disease-causing variants that are more resistant to the effects of vaccines emerge. The rapid genetic mapping of the agent at the beginning of the COVID-19 pandemic has led to the rapid initiation and development of vaccine studies with technological infrastructure preparation. Although being updated rapidly, as of June 2021, there are a total of 12 vaccines, 11 of which are approved for emergency use and licensed, and one vaccine is about to be approved for use World Health Organization (2021). COVID-19 vaccine tracker and landscape [online]. Website https://www.who.int/publications/m/item/draft-landscape-of-covid-19-candidate-vaccines [accessed 15 June 2021].. Although the criteria used to determine the efficacy of vaccines differ between studies, it has been shown that the rate of disease spread decreased, and severe disease was prevented with the vaccines in use The Institute for Health Metrics and Evaluation (IHME) global health research (2021). COVID-19 vaccine efficacy summary [online]. Website http://www.healthdata.org/covid/covid-19-vaccine-efficacy-summary [accessed 15 June 2021 ]. [29]. Every country has made an effort to access the vaccines approved for emergency use within the framework of the rules determined by the health authority. Countries that can rapidly produce their vaccines using their own technology are the luckiest in this regard. USA; Pfizer/BioNTech, Moderna (m-RNA vaccines), J&J vaccines; Israel Pfizer/BioNTech vaccine; and Gulf Countries through the widespread use of Pfizer/BioNTech and Sinopharm vaccines have succeeded in reducing the number of new cases and removing the use of masks in outdoor conditions from routine practice. In Turkey, the subjects of vaccine supply and vaccine production have been discussed rapidly. An inactivated virus vaccine (CoronaVac) produced by a Chinese manufacturer (Sinovac) has been tested in Phase III studies in Brazil, Turkey, Indonesia, China, and Chile. The efficacy of the vaccine was evaluated in a placebo-controlled Phase III study performed on more than 10,000 volunteers aged 18–59 years in 24 centers in Turkey. The vaccine was administered in two doses at 0–14 days. It has been shown that 14 days after the second dose, the vaccine’s protection against symptomatic disease is 83.5% and 100% in preventing severe disease requiring hospitalization [30]. Afterward, the Sinovac vaccine application in the community was started on January 13, 2021. Currently, also with the introduction of the Pfizer/BioNTech vaccine, the vaccine administration rate has exceeded one million per day. With such a high vaccination rate, Turkey has been appreciated by the WHO Republic of Turkey Ministry of Health (2021) [online]. Website https://www.saglik.gov.tr/TR,84147/dso-avrupa-direktorunden-turkiyeye-tebrik.html [accessed 15 June 2021] .

### 1.6. Accurate information, healthy data sharing, and effective field studies

It was an infodemic that we encountered intensely with the COVID-19 pandemic. In the early period of the epidemic, WHO created a website on this subject and announced the wrong information, which caused it to become dominant and to put the communities in a panic mood, with the correct answers World Health Organization (2021). COVID-19 Mythbusters-WHO [online]. Website https://www.who.int/emergencies/diseases/novel-coronavirus-2019/advice-for-public/myth-busters [accessed 15 June 2021].. In Turkey, the Ministry of Health created a web page for COVID-19 and tried to deliver all up-to-date information to the public on time and with correct content Republic of Turkey Ministry of Health (2021). COVID-19 information page [online]. Website https://covid19.saglik.gov.tr [accessed 15 June 2021]. [31]. Studies have shown that in the fight against the pandemic, providing the right information to the communities clearly and understandably leads to an increase in compliance with the measures, accelerates the vaccination studies, and reduces the public concerns about the epidemic [32–34]. Being in constant communication, providing cooperation between the health authorities of the countries and professional associations solve the health crises effectively and quickly.

It should not be forgotten that epidemic control will be achieved on the field. While clinicians follow COVID-19 cases, epidemiologists will simultaneously ensure that the pandemic is kept under control in the community. Epidemiological studies may seem time-consuming and costly, especially in epidemic situations however, control can be maintained by well-defined surveillance studies, epidemiological studies, and international cooperation to be carried out actively in the field [35].

### 1.7. Lockdown strategies

#### 1.7.1. Lockdown strategies

Governments around the world have adopted varying prevention strategies. Increased testing frequency to detect asymptomatic carriers, symptom-based screening and self-isolation, quarantine, and complete lockdown were among these social distancing strategies (Table 1). 

Within the context of this pandemic, lockdown can be defined as a set of mandatory and indiscriminate measures with the aim of reducing transmission of COVID-19 that involve limitations on social and economic life. The most common usage of the term lockdown during the COVID-19 pandemic was for mass quarantines or stay-at-home orders. The first lockdown during the COVID-19 pandemic was implemented in Wuhan on January 23, 2020 [36]. Many governments around the World have declared national emergencies and implemented lockdowns of varying extents to enforce social distancing as a response to the spread of the disease. While lockdowns limit movements or activities in a population, they may allow some organizations (especially the ones that supply basic needs and services) to function normally.

It has been shown that lockdowns are effective in exponentially slowing down the spread of SARS-CoV-2 in terms of infection rate and death [37–40]. Following the implementation of an effective lockdown, the number of new COVID-19 infections starts to reduce after approximately 10 days and continues to reduce up to 20 days after the implementation [37].

Lockdown strategies to control COVID-19 are irrefutably sophisticated due to having a wide range of beneficial and harmful effects related to public health, social interactions, and economics. Moreover, the benefits and harms of the lockdown measures are different across the country. Lockdowns had dramatically changed people’s daily lives with deep consequences. Increases in anxiety and depression in affected communities may be expected. 

#### 1.7.2. Lockdown in Turkey 

**Figure 2:**
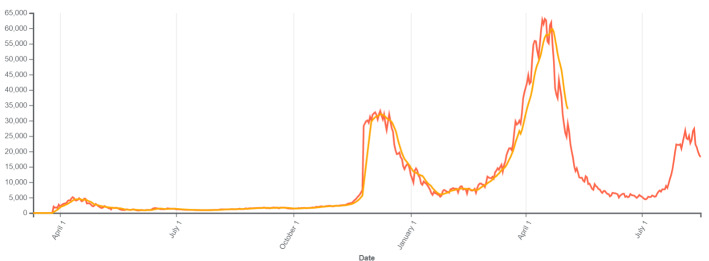
Effect of lockdown in Turkey.

Several different containment measures were implemented by the Turkish government. These included complete lockdown and other social distancing strategies (Table 2). In Turkey, lockdown measures were taken by the government on 4 May 2020, 20 November 2020, and 29 April 2021, upon the increase in the number of cases. With these measures, the number of cases decreased (Figure 2) Wikipedia (2020). COVID-19 Pandemic in Turkey [online]. Website https://en.wikipedia.org/wiki/COVID-19_pandemic_in_Turkey [accessed 28 June 2021]..

**Table 2:** Restriction measures in COVID-19.

Prohibition of the general population from leaving their homes (lockdown).
Prohibition of the local population (at least in one region) from leaving their homes (local-lockdown).
Encouragement of voluntarily staying at home.
Encouragement of risk groups or vulnerable groups such as the elderly to stay at home.
Restrictions on gatherings or implementation of interventions to limit all public/private, indoor/outdoor gatherings.
Closures of schools or other educational or daycare institutions.
Mandatory mask use in public spaces.
Closure of businesses or other public places (restaurants, bars, theatres, public transport, etc.).
Closure of accommodation businesses (hotels etc.).
Closure of places of worship.
Encouragement of remote working.
Workspace modifications to reduce transmission risk.

In conclusion, COVID-19 has taught us the lesson that “World Health” is necessary. Medicine and health organizations should always cooperate. Information sharing is very important. Explicit sharing of the data with society is a factor in increasing harmony. It is always important for all “World Health”, especially for pandemic management, to put into practice tested approaches to epidemics, to rely on shared data and information. The value of institutions and countries helping countries with limited resources and working together in the spirit of global health was once again understood with the COVID-19 pandemic.
